# Nurse case management to improve multidrug-resistant TB: a cluster-randomised trial

**DOI:** 10.5588/ijtldopen.26.0126

**Published:** 2026-07-13

**Authors:** J.E. Farley, K. Lowensen, C. Budhathoki, A. Leonard, K. McNabb, C.M. Weizer, L. Krotee, A.J. Bergman, N.N. Ndjeka

**Affiliations:** 1 Johns Hopkins University School of Nursing, Baltimore, MD, USA;; 2 Johns Hopkins Center for Infectious Disease and Nursing Innovation (CIDNI), Baltimore, MD, USA;; 3 Johns Hopkins Center for AIDS Research (CFAR), Baltimore, MD, USA;; 4 Johns Hopkins Center for Tuberculosis Research (TRAC), Baltimore, MD, USA;; 5 TB Control and Management, Department of Health, Pretoria, South Africa.

**Keywords:** tuberculosis, South Africa, MDR-TB outcomes, treatment, nursing, chronic care model

## Abstract

**BACKGROUND:**

Nurse case management (NCM) models have improved outcomes in chronic disease and HIV, but evidence in multidrug-resistant TB (MDR-TB) is limited. We therefore evaluated whether NCM could improve MDR-TB outcomes.

**METHODS:**

This pragmatic, cluster-randomised trial was conducted at 10 district hospitals in KwaZulu-Natal and Eastern Cape, South Africa.

**RESULTS:**

Between 13 November 2014 and 5 September 2019, 2,844 were enrolled. A total of 2,134 were analysed (1,093 NCM, 1,041 usual care). Among 1,236 men (57.9%) and 898 women (42.1%), mean age was 37.4 years (standard deviation 12.2). Treatment success occurred in 706 (64.5%) of NCM group and 645 (61.9%) in usual care (*P* = 0.62). Treatment failure occurred in 28 (2.6%) of NCM group and 47 (4.5%) of usual care. NCM did not improve the proportion of successful treatment (adjusted odds ratio [aOR] = 0.79, *P* = 0.17, and 95% confidence interval [CI]: 0.56, 1.12). In secondary analysis, NCM reduced the odds of treatment failure by 44% (aOR 0.56, 95% CI: 0.31–0.99), which improved to 49% (aOR 0.51, 95% CI: 0.26–0.99) in sensitivity analysis. Differences for other outcomes were not significant.

**CONCLUSION:**

NCM did not improve MDR-TB treatment success. Yet, NCM was associated with reduced odds of treatment failure. The intervention alone was insufficient to overcome barriers associated with loss to follow-up, or delayed presentation.

Nurse case management (NCM) models involve the systematic evaluation and delivery of evidence-based health services and care coordination designed to improve clinical outcomes. In such models, care is typically initiated by a physician and NCM supports a comprehensive package of supportive services. While these models have shown great promise in increasing patient-centred care for both chronic disease management and HIV,^1,2^ there is less evidence for TB.^3^ A recent scoping review detailing patient-centred TB models of care identified only one published experimental design between 2006 and 2019, which focused on stigma reduction.^4^

Despite the limitations in the published drug-resistant TB literature, patient-centred care is a hallmark of the WHO End TB Strategy.^5^ Patient support and care coordination efforts between TB and HIV clinical services are exemplars noted within the strategy. As demonstrated in the systematic review and meta-analysis by Yan and Bai,^6^ interventions addressing optimised nursing care for people with TB has been shown to improve adherence to treatment, sputum conversion, patient satisfaction, social function, and mental health. These interventions, however, are narrowly focused on one aspect of care (e.g., adherence or education).^6^ Such approaches fail to address patient, systems, and structural barriers to care that are often noted by patients in the qualitative literature.^7^ There are no randomised trials describing patient-centred NCM as a care coordination model to improve TB treatment outcomes for multidrug-resistant TB (MDR-TB).

Until the recent advances in treatment regimens, MDR-TB required between 9 and 24 months of antimicrobial treatment. In South Africa, this treatment regimen consisted of a 6-month daily injectable aminoglycoside-based intensive phase, followed by an all-oral regimen for up to 18 months. The treatment burden on the patient was significant. The complex nature of this care model necessitates a cadre of skilled workers who can navigate the health system structure, triage patient-related adverse events between visits, coordinate clinical encounters, and provide both psychosocial and medical adherence counselling. The objective of this trial was to evaluate a multifaceted, nurse-led case management intervention for people newly diagnosed with MDR-TB in South Africa through a 5-year, cluster-randomised trial (CRT). Despite the movement away from injectable aminoglycoside regimens to all-oral short course therapy in recent years, the lessons from this trial are illustrative, as health system fragmentation, adverse events, and limited patient-centred approaches to care remain.

## METHODS

This trial utilises a pragmatic CRT design. Ten district hospitals in the provinces of Eastern Cape and KwaZulu-Natal, South Africa, were randomised. Participants were enrolled between 13 November 2014 and 5 September 2019, and followed through treatment outcome, which occurred for the last patient on 10 March 2021. We compared our NCM intervention in five hospitals to usual care (UC) in five control sites. MDR-TB treatment was provided by the National Department of Health of South Africa per guideline-based, programmatic management.

### Study setting and cluster eligibility

Among the 10 hospitals, six were in KwaZulu-Natal in the districts of eThekwini, Ugu, uMgungundlovu, uMkhanyakude, and Harry Gwala. The remaining four were in the Eastern Cape in the districts of Nelson Mandela, Sara Baartman, and Buffalo City. To be eligible, MDR-TB hospitals must provide care in accordance with national clinical guidelines for at least 6 months prior to trial enrolment, have access on-site to anti-retroviral therapy (ART), and be willing to participate. Standardised programmatic management for MDR-TB (i.e., weight-based dosing of a standard regimen, set follow-up intervals, as well as adverse event monitoring recommendations) was implemented at each site and updated to remain consistent with national guidelines throughout the trial.

### Participant eligibility

A person ≥13 years of age, with microbiologically confirmed MDR-TB who received care at a participant site and signed consent (or assent with consent by a legally authorised representative) within 7 days of treatment initiation was eligible. A person was eligible regardless of whether their MDR-TB treatment was started as an inpatient or an outpatient. Children <13 years old, those enrolled in another clinical trial, persons requiring individualised MDR-TB treatment regimens (e.g., extremely drug-resistant TB [XDR and pre-XDR]), persons with known extra-thoracic TB, or persons unwilling or unable to provide consent were excluded. Participants were recruited at treatment initiation visits.

### Cluster randomisation and masking

We randomised the participating hospitals (clusters) to either NCM intervention group or a UC control group. We used a highly restricted randomisation approach to achieve a close balance across arms on the following variables: MDR-TB programme size (i.e., 300 or more patients per year vs. 299 or less per hospital/clinic/cluster on average) as determined the year prior to trial launch and geographical location (i.e., rural vs. urban/peri-urban) of the hospital.^8^ Within each of the strata, the trial statistician assigned each hospital or cluster to either the intervention or control arm in a one-to-one allocation prior to enrolment. The randomisation plan was generated using an SAS procedure. Masking of participants and intervention staff was not feasible due to the nature of the intervention.

### Nurse case management (intervention)

The development and pilot testing of the intervention has been previously published.^3^ The intervention utilised the theoretical domains of the Chronic Care Model with added attention to linkage to care, building on evidence-based guidelines and our team’s pilot findings.^9^ Supplementary Data Table S1 details the features of the NCM intervention model. Nurse case managers were professional 3-year diploma or 4-year-degree nurses and provided enhanced case management training to improve patient adherence, psychosocial support, adverse event detection and monitoring, disease management, and clinical quality for patients with MDR-TB and HIV. The intervention was not designed to alter patient interactions with clinicians except to ensure clinician guideline-based care adherence, and to streamline the evaluation through weekly and monthly goal monitoring. Competency was assessed post NCM training through direct observation of intervention implementation and annual updates to refresh knowledge and skills. The planned nurse-to-patient ratio was one nurse caring for 300 participants over the entire 5-year trial period, with enrolment targets of approximately 100 participants per year. The panel was built slowly, with new participants being admitted to the MDR-TB hospitals at a rate of 10–15 per month, until the recruitment goals for the year were reached. The frequency of interaction was planned to be two to three times per week during the intensive phase of treatment (6–8 months) and monthly during the continuation phase.

### Usual care (control)

UC is defined as standardised programmatic management of MDR-TB and HIV without care coordination. Nurses are part of the team, but they do not have a special coordination role, leaving the MDR-TB physician solely responsible for treatment outcomes with little support outside of brief adherence education and the collection of required laboratory investigations. Specialist MDR-TB physicians and nurses see patients weekly during the intensive phase, and the patient receives basic daily nursing care while inpatient without coordination of care, nor active monitoring of adverse drug reactions or HIV care integration. This care transitioned to monthly physician visits in the continuation phase.

### Primary outcomes

MDR-TB treatment outcome was the primary outcome and was defined as the proportion of patients experiencing treatment success (i.e., a combination of cure and treatment completion) by arm. Both the South African National Department of Health and this trial utilised the WHO definitions for TB treatment outcome. These definitions were modified most recently by the WHO in 2022^10^ and these definitions have been applied to this dataset. Briefly, a treatment success is defined as either evidence of a microbiologic cure or treatment completion without all the necessary culture results for cure. Negative outcomes include lost to follow-up during treatment, death for any reason during treatment, treatment failure through intolerance to treatment, or worsening resistance profile. We excluded participants who transferred their care to another treatment facility prior to completion of MDR-TB treatment. The central hypothesis was that NCM in intervention sites will increase MDR-TB cure and completion rates (i.e., treatment success) in comparison to UC, that is, standardised programmatic management alone, in patients with and without HIV co-infection.

### Cluster size determination

To determine the number of clusters necessary to achieve 80% power, we selected an intra-class correlation (ICC) of 0.05. This low ICC was based on our prior work within these facilities, where we identified similar treatment outcomes across provinces and hospitals.^11^ We believed this was related to the standardised management throughout the country, which limited between facility treatment variations. We determined that 1,500 subjects per assignment and 300 subjects in each cluster would be needed given the ICC of 0.05 and a total of 10 clusters (five intervention and five control). The control group success proportion was assumed 0.60, based on recent data from Department of Health collaborators since the increase in availability of ART. The intervention group proportion was assumed to be 0.60 under the null hypothesis and 0.80 under the alternative hypothesis (effect size 0.18; providing 88% power). The test statistic would be a two-sided Wald test from a generalised estimating equation (GEE) log-binomial model for treatment success at 24 months post-enrolment with α = 0.05.

### Statistical analysis

The main independent variable was trial arm. The main dependent variable was treatment outcome, pre-specified as treatment being successful or unsuccessful. We also analysed the multinomial outcome with multiple categories including death; loss to follow-up; treatment failure; and treatment success (cure/treatment completion). We summarised demographic and baseline variables by trial arm. The differences on demographics and baseline variables between the arms were tested by taking patient clustering within hospital into account using GEE. This allowed assessment of balance between the arms on the key demographic and baseline variables. This analysis also identified potential covariates, in addition to clinically meaningful covariates, to be included in adjusted analyses to compare the outcome variable between the arms. A logit link for dichotomous variables, generalised logit link for multicategory categorical variables, and an identity link for continuous variables were used in the GEE models. The primary outcome analysis used binary GEE modelling with a logit link and independent correlation structure to determine the effects of NCM on final trial outcomes. The secondary outcome analysis used multinomial GEE modelling with a generalised logit link and independent correlation structure to compare NCM and UC groups on the multicategory outcomes. We first fit a bivariate GEE model for evaluating relationship between the outcome and each of the predictor variables or covariates. We then constructed a multivariable GEE model considering age, sex, marital status, body mass index (BMI), comorbidity, home internet (as a proxy measure of income), adequate food availability (as a proxy measure of income), baseline smear status, baseline culture status, and regimen type (two-categories) as potential covariates. These clinical variables were selected a priori based on their clinical relevance, while home internet availability and adequate food were included as proxy indicators of socio-economic status. The final models (binary and multinomial) were selected based on stepwise forward method using the quasi-likelihood under independence criterion. The odds ratios (ORs) and 95% confidence intervals (95% CIs) quantifying the relationships between the intervention and the outcome were reported. The patient outcome evaluation followed a modified intent-to-treat approach that used all the available observations with trial treatment eligible patients, with a regression imputation using maximum likelihood method for BMI in the setting of missing height or weight. All data management and statistical analyses were performed using SAS software version 9.4.

### Ethical statement

This trial was approved by the Johns Hopkins University School of Medicine (IRB# NA_00078899), the University of KwaZulu-Natal (Application #BE530/14), as well as the Provincial Research Committees in the Eastern Cape and KwaZulu-Natal. The trial protocol is available at ClinicalTrials.gov (https://clinicaltrials.gov/study/NCT02129244).

## RESULTS

The trial enrolled 2,844 participants with 2,134 included in the final analysis. Among these, 1,093 were assigned to the NCM arm and 1,041 to the UC arm. The rationale for exclusion of participants is noted in the CONSORT diagram (Figure). The socio-demographic, social, and clinical characteristics are presented in Table 1. Among the 2,134, the mean age of the sample was 37.4 years (standard deviation [SD] 12.2), 1,236 were male (57.9%), 1,232 were single (57.7%), 1,250 were unemployed (58.6%), and 1,561 had some secondary education or less (73.1%). Just over half, 1,085 (50.8%) had a documented prior history of TB infection, and, among those with a baseline chest X-ray, 999 (63.5%) had bilateral disease. Most (1,502) participants (70.4%) received an injectable-based regimen for MDR-TB treatment. The majority (1,583) participants were living with HIV (74.2%), and while 1,126 (71.1%) had exposure to ART, only 478 (30.2%) had viral suppression <200 copies/mL on enrolment.

**Figure.**
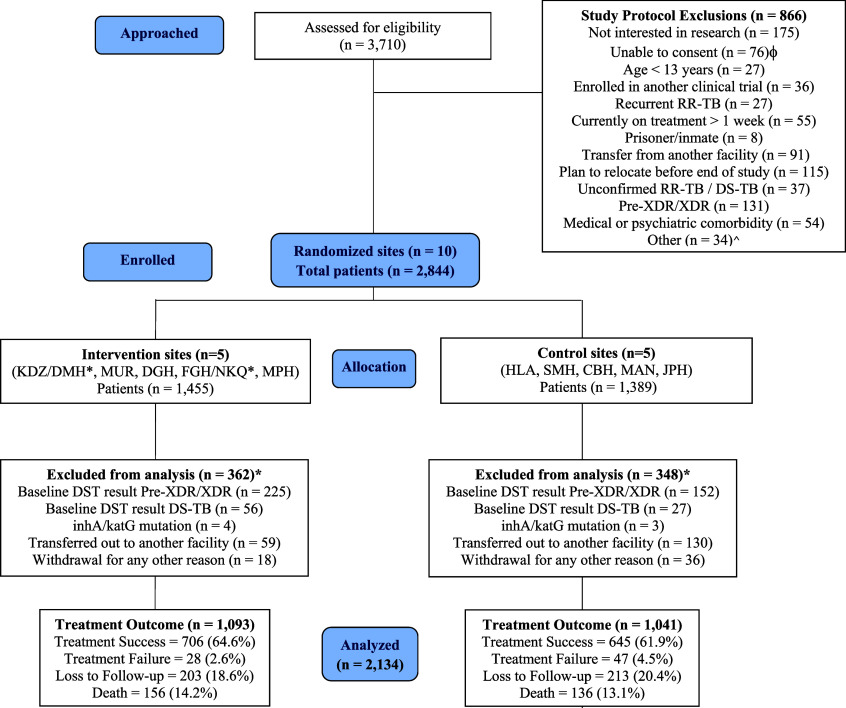
Nurse case management CONSORT diagram. ϕ Unable to consent includes the following: unable to participate in the consent process, unable to consent with any of the approved languages in consent forms or all screening laboratory results were not available prior to consent. ^ Other includes: 2 INH resistance; 2 second-line mutations; 21 admissions for Bedaquiline initiation; 7 Non-standard regimen; 1 No contact details/phone; 1 No lab results. *KDZ transferred all patients to DMH as they became an XDR-TB hospital only and FGH closed completely and all patients were transferred to NKQ.

**Table 1.** Sample socio-demographic, social, and clinical characteristics.

Characteristics	Total, N = 2,134	NCM intervention, N = 1,093	Control, N = 1,041
Socio-demographics			
Age (year), mean (SD)	37.4 (12.2)	38.2 (12.4)	36.5 (12.0)
Sex, n (%)			
Female	898 (42.1)	435 (39.8)	463 (44.5)
Male	1,236 (57.9)	658 (60.2)	578 (55.5)
Residential area, n (%)			
City/CBD/suburb	80 (3.7)	50 (4.6)	30 (2.9)
Farm	20 (0.9)	15 (1.4)	5 (0.5)
Township	765 (35.8)	540 (49.4)	225 (21.6)
Rural village	1,255 (58.8)	479 (43.8)	776 (74.5)
Unknown	14 (0.7)	9 (0.8)	5 (0.5)
Housing type, n (%)			
House	1875 (87.8)	954 (87.3)	921(88.4)
Flat	53 (2.5)	27 (2.5)	26 (2.5)
Informal/shack	160 (7.5)	96 (8.8)	64 (6.2)
Homeless/other**^A^**	46 (2.2)	16 (1.4)	30 (2.9)
Highest level of education, n (%)			
None/less than secondary	1,561 (73.1)	790 (72.3)	771 (74.1)
Secondary school	415 (19.4)	218 (19.9)	197 (18.9)
Some technical/college training	75 (3.5)	45 (4.1)	30 (2.9)
Technical/college completed	83 (3.9)	40 (3.7)	43 (4.1)
Employment status, n (%)			
Employed	661 (31.0)	373 (34.1)	288 (27.7)
Student	142 (6.6)	59 (5.4)	83 (8.0)
Unemployed	1,250 (58.6)	615 (56.3)	635 (61.0)
Retired/disabled	69 (3.2)	37 (3.4)	32 (3.1)
Unknown	12 (0.6)	9 (0.8)	3 (0.2)
Marital status category, n (%)			
Married	252 (11.8)	148 (13.5)	104 (10.0)
Partnered/in a relationship	536 (25.1)	182 (16.7)	354 (34.0)
Separated/widowed	97 (4.5)	30 (2.7)	67 (6.4)
Single	1,232 (57.7)	722 (66.1)	510 (49.0)
Unknown	17 (0.8)	11 (1.0)	6 (0.6)
History of TB, n (%)			
No/not documented	1,049 (49.2)	524 (47.9)	525 (50.4)
Yes	1,085 (50.8)	569 (52.1)	516 (49.6)
Adequate food available daily, n (%)			
Yes	1,564 (73.3)	932 (85.3)	632 (60.7)
No/unknown	570 (26.7)	161 (14.7)	409 (39.3)
Ever incarcerated, n (%)			
No/unknown	1886 (88.4)	973 (89.0)	913 (87.7)
Yes	248 (11.6)	120 (11.0)	128 (12.3)
Government finance assistance, n (%)			
No/unknown	1809 (84.8)	978 (89.5)	831 (79.8)
Yes	325 (15.2)	115 (10.5)	210 (20.2)
Illicit drugs ever, n (%)			
No/unknown	1943 (91.0)	1,008 (92.2)	935 (89.8)
Yes	191 (9.0)	85 (7.8)	106 (10.2)
Smoker (lifetime),**^B^** n (%)			
No/unknown	1,391 (65.2)	744 (68.1)	647 (62.1)
Yes	743 (34.8)	349 (31.9)	394 (37.9)
Any alcohol use, n (%)			
No/unknown	1,273 (59.6)	681 (62.3)	592 (56.9)
Yes	861 (40.4)	412 (37.7)	449 (43.1)
Internet at home, n (%)			
No/unknown	1717 (80.5)	937 (85.7)	780 (74.9)
Yes	417 (19.5)	156 (14.3)	261 (25.1)
Clinical characteristics			
Baseline chest X-ray, n (%)			
Yes	1,572 (73.7)	842 (77.0)	730 (70.1)
No/not documented	562 (26.3)	251 (23.0)	311 (299)
Chest baseline radio extent, n (%)	N = 1,572	N = 842	N = 730
Normal	74 (4.7)	24 (2.8)	50 (6.8)
Unilateral	347 (22.1)	151 (17.9)	196 (26.8)
Bilateral	999 (63.5)	619 (73.5)	380 (52.2)
Abnormal, sides unknown or not documented	152 (9.7)	48 (5.7)	104 (14.2)
Chest baseline cavity, n (%)	N = 1,470	N = 782	N = 688
No cavity	665 (45.2)	391 (50.0)	274 (39.8)
Unilateral	271 (18.4)	129 (16.5)	142 (20.6)
Bilateral	179 (12.2)	103 (13.2)	76 (11.0)
Sides unknown or not documented	355 (24.2)	159 (20.3)	196 (28.5)
BMI	N = 2,134	N = 1,093	N = 1,041
BMI (imputed), mean (SD)	20·6 (4.6)	20·9 (4.9)	20·3 (4.2)
BMI (imputed), category, n (%) (≥20 years only)	N = 2034	N = 1,057	N = 977
Underweight	661 (32.5)	345 (32.6)	316 (32.3)
Normal	1,100 (54.1)	536 (50.7)	564 (57.7)
Overweight	180 (8.8)	116 (11.0)	64 (6.6)
Obese	93 (4.6)	60 (5.7)	33 (3.4)
Comorbidity (non-HIV), n (%)			
No/unknown	1890 (88.6)	944 (86.4)	946 (90.9)
Yes	244 (11.4)	149 (13.6)	95 (9.1)
MDR-TB provider initiating treatment, n (%)			
Physician	1929 (90.4)	888 (81.2)	1,041 (100.0)
Nurse	200 (9.4)	200 (18.3)	0 (0.0)
Clinical associate	5 (0.2)	5 (0.5)	0 (0.0)
Baseline smear status, n (%)			
Negative	1,146 (53.7)	623 (57.0)	523 (50.2)
Positive	915 (42.9)	432 (39.5)	483 (46.4)
Not collected or contaminated/leaked	73 (3.4)	38 (3.5)	35 (3.4)
Baseline culture status, n (%)			
Negative	640 (30.0)	356 (32.6)	284 (27.3)
Positive	1,210 (56.7)	606 (55.4)	604 (58.0)
Not collected or contaminated/leaked	284 (13.3)	131 (12.0)	153 (14.7)
Regimen (two-categories), n (%)			
All oral	632 (29.6)	394 (36.1)	238 (22.9)
Injectables/Inj to BDQ	1,502 (70.4)	699 (63.9)	803 (77.1)
HIV, n (%)			
No/not tested/unknown	551 (25.8)	268 (24.5)	283 (27.2)
Yes	1,583 (74.2)	825 (75.5)	758 (72.8)
HIV ART ever, n (%)			
No/not reported	457 (28.9)	255 (30.9)	202(26.7)
Yes	1,126 (71.1)	570 (69.1)	556 (73.3)
CD4 count category, n (%)	N = 1,583	N = 825	N = 758
<200	750 (47.4)	411 (49.8)	339 (44.7)
200–500	458 (28.9)	233 (28.2)	225 (29.7)
>500	215 (13.6)	120 (14.5)	95 (12.5)
Missing	160 (10.1)	61 (7.4)	99 (13.1)
Viral load category, n (%)	N = 1,583	N = 825	N = 758
>200: Detectable	631 (39.9)	317 (38.4)	314 (41.4)
<=200: Undetectable	478 (30.2)	231 (28.0)	247 (32.6)
Unknown VL or not done	474 (29.9)	277 (33.6)	197 (26.0)

BMI categories are presented only for 20 years or older. For patients 13 and <20 years, we would need percentiles to determine categories, and the percentiles calculation needs head circumference per CDC guidelines. No head circumference was measured for the adolescents in this study. Non-HIV comorbidities: Hypertension, arrhythmia, diabetes, thyroid disorder, liver disease, and kidney disease.

ART = anti-retroviral treatment; BDQ = bedaquiline; BMI = body mass index; CBD = central business district; Inj = injection; MDR-TB = multidrug-resistant TB; NCM = nurse case management; VL = viral load.

A Other: rental, hostel, government house, park home, workshop, and unknown.

B A patient who used >100 cigarettes, cigars or pipefuls in their lifetime was considered to be a smoker in this study.

Among trial participants, we identified overall treatment success among 1,351 (63.3%), slightly higher in the intervention (706, 64.5%) compared to control (645, 61.9%), however, this difference was not statistically significant in the bivariate model (Table 2). Loss to follow-up occurred among 416 (19.5%) of participants, again slightly less in the intervention (203, 18.6%) compared to control (213, 20.5%); this difference was also not statistically significant. There were 292 deaths (13.7%), with fewer deaths seen in the control (136, 13.1%) compared to the intervention (156, 14.3%), a difference that was again not statistically significant. Treatment failure was identified among 75 (3.5%) participants, statistically lower in the intervention (28, 2.6%) compared to control (47, 4.5%).

**Table 2.** Multidrug-resistant TB treatment outcomes by study arm, primary bivariate, and secondary multicategory analysis.

Characteristics, n (%)	Total (n = 2,134)	Int group (n = 1,093)	UC group (n = 1,041)	*P* value
Primary outcome (dichotomous)				
Treatment successful	1,351 (63.3)	706 (64.5)	645 (61.9)	REF
Treatment unsuccessful (negative outcomes)	783 (36.7)	387 (35.4)	396 (38.1)	0.62
Secondary outcome (multicategory)				
Success	1,351 (63.3)	706 (64.5)	645 (61.9)	REF
Died	292 (13.7)	156 (14.3)	136 (13.1)	0.87
Loss to follow-up	416 (19.5)	203 (18.6)	213 (20.5)	0.62
Treatment failure	75 (3.5)	28 (2.6)	47 (4.5)	0.04

For the primary multivariable GEE analysis using a binary regression model, we found that the difference between the arms on treatment positive outcomes was not statistically significant (aOR = 0.79, *P* = 0.182, 95% CI: 0.56, 1.12; Table 3). In a secondary analysis with multinomial regression model, we found that NCM was successful in reducing MDR-TB treatment failure by 44% (aOR: 0.56, 95% CI: 0.31–0.99) compared to UC. NCM did not have an impact on treatment success, loss to follow-up, or death (Table 3). In a sensitivity analysis, we modelled the impact of clinician-determined cavitary disease as a marker of disease severity among a smaller sample of patients (N = 1,470) with baseline chest X-ray results available. We found NCM in this model to again reduce the odds of treatment failure, this time by 49% (aOR: 0.51, 95% CI: 0.26–0.99) compared to UC (Table 4).

**Table 3.** Main trial outcome (success vs. unsuccessful) and secondary multicategory outcome.

Outcome (N = 2,134)	Bivariate			Multivariable (M)		
	OR	*P* value	95% CI	aOR	*P* value	95% CI
Dichotomous						
Unsuccessful	0.89	0.62	0.57, 1.40	0.79	0.18	0.56, 1.12
Successful (REF)	–	–	–	–	–	–
Multicategory						
Died	1.05	0.87	0.61, 1.81	0.84	0.417	0.56, 1.27
LTFU	0.87	0.62	0.50, 1.50	0.81	0.4001	0.50, 1.32
Tx failure	0.54	0.04	0.31, 0.96	0.56	0.046	0.31, 0.99
Success (REF)	–	–	–	–	–	–
QIC	4,302.3414 (N = 2,134)			4,232.7405 (N = 2,134)		

The analysis was conducted using generalised estimating equations. The covariates used in the multicategory model for the adjusted analyses were age, sex, marital status, comorbidity, home internet, and adequate food availability. The following covariates were considered in multivariable analysis: age, sex, marital status, body mass index, comorbidity, home internet, adequate food availability, baseline smear status, baseline culture status, and regimen type (three-categories and two-categories).

OR = odds ratio (bivariate); aOR = adjusted OR (multicategory); CI = confidence interval; LTFU = lost to follow-up; Tx = treatment; QIC = quasi-likelihood under independence criterion.

**Table 4.** Sensitivity analysis with cavity on baseline chest X-ray to final multicategory model.

Outcome	Multivariable M + baseline cavity (N = 1,470)		
	aOR	*P* value	95% CI
Dichotomous			
Unsuccessful	0.79	0.25	0.53, 1.18
Successful (REF)	–	–	–
QIC			
Multicategory			
Died	1.02	0.94	0.67, 1.54
LTFU	0.71	0.24	0.40, 1.25
Tx failure	0.51	0.05	0.26, 0.99
Success (REF)	–	–	–
QIC	2,809.486 (n = 1,470)		

The covariates were age, sex, marital status, comorbidity, home internet, and adequate food availability, and chest baseline cavity.

LTFU = lost to follow-up; Tx = treatment; QIC = quasi-likelihood under independence criterion; aOR = adjusted odds ratio; CI = confidence interval.

## DISCUSSION

Although treatment success was higher in the NCM intervention arm, we did not find a significant intervention effect. The pragmatic nature of the trial limited the intervention to available resources within the public system of care and community-specific resources differed by site. Late presentation to care was a hallmark of death in the trial. On enrolment, participants demonstrated multiple clinical features associated with late presentation, including low BMI (mean 20.6, SD 4.6), extensive bilateral disease on chest X-ray, along with CD4 count <200 (47.4%) and poor viral suppression (30.2%) among those on ART. After treatment initiation, failure to achieve viral suppression during MDR-TB treatment has been demonstrated as a frequent reason for poor treatment outcomes^12,13^ and HIV resistance testing is rare.

In our secondary multinomial evaluation, which controlled for the imbalance between arms, we found that participants receiving treatment at hospitals with NCM experienced a 44% reduction in the odds of treatment failure. The odds further improved to a 49% reduction among those with a baseline chest X-ray available, which included the extent of baseline cavitary disease. While the meta-analysis by Yan and Bai^6^ demonstrated impact of nursing-specific interventions, these prior studies each evaluated the impact of a nursing practice intervention to improve process measures (i.e., treatment adherence or satisfaction) or mental health outcomes (i.e., anxiety or depression). None of these studies offered insights into the impact on TB disease outcomes, so our finding is hypothesis generating related to the mechanisms at work to reduce treatment failure. We believe, adverse event monitoring, as part of the Chronic Care Model decision support domain, coupled with adherence counselling and support, as part of the patient self-support domain, was key to this finding. This finding offers support to WHO recommendations to implement patient-centred care models for MDR-TB treatment.^10,14^ Upon identification of adverse events, nurses were able to provide counselling to mitigate their symptoms and/or link participants to clinicians to prescribe appropriate treatment to improve the event, as needed. We theorise that this component of the intervention (i.e., supporting easier access to adjuvant treatments to mitigate treatment-related adverse events) is likely the mechanism driving this effect.

It is possible that, despite improved adherence to treatment for both MDR-TB and HIV, the clinical outcome was driven by unmeasured HIV treatment resistance. Prior sub-group analysis of those successfully completing treatment within this trial demonstrated that despite clinical evidence of adherence to MDR-TB treatment, lack of viral suppression at treatment completion was associated with negative outcomes.^12,13^ Among the treatment arms, we did find a statistically greater unknown viral load in the intervention arm (33.6%), meaning no viral load within 12 months preceding MDR-TB treatment and no viral load obtained by the clinician within the first 3 months of MDR-TB treatment. Future NCM studies in populations with high HIV co-infection should prioritise viral load monitoring throughout treatment and ongoing adherence to ART during MDR-TB treatment.

NCM was not shown to be effective in preventing loss to follow-up. Loss to follow-up is multifaceted,^14^ with poverty as a key driving mechanism. The NCM intervention was unable to provide financial support to overcome barriers to care, such as transportation, loss of employment, and informal income associated with MDR-TB treatment. McNabb et al.^15^ previously evaluated distance to treatment among participants in this trial and found the median travel distance was 40.96 km (interquartile range: 17.12, 63.49). In this nested cohort, travel distance > 60 km increased the odds of loss to follow-up by 91%.^10^ The intervention was unable to provide routine home visitation, which may have helped reduce loss to follow-up.

This trial has several limitations. First, the trial may have been underpowered to see the true impact of the intervention. Although the trial reached the targeted sample of 3,000 enrolled participants, we excluded 347 individuals from this analysis due to pre-specified exclusions that could not be identified at enrolment, namely the presence of resistance indicating pre-XDR/XDR. The patient population was also more transient than anticipated and we experienced a higher than anticipated transfer from our trial sites to a facility not participating in the trial. Second, the enrolment period for the trial was longer than anticipated. As such, multiple MDR-TB treatment changes occurred during the trial, namely shortening of the 24-month regimen initially to 18 months, then to 9 months with the introduction of newer drugs. Regimen type (oral 9-month regimen compared to 18- or 24-month injectable regimens) was controlled for in the GEE models to account for these differences. In evaluating balance between the intervention and control arms of the trial, we identified statistical differences in several key clinical variables that may have influenced death. For instance, participants in the intervention arm were statistically more likely to be male, be diagnosed with a chronic disease comorbidity, and have bilateral disease on chest X-ray, which have been independently shown to be associated with increased mortality in TB patients.^16–18^ Participants in the control arm were statistically more likely to have adequate food daily and have internet available at home, marker of higher socio-economic status. These imbalances may have arisen from the nurse case managers being more focused on recruitment of participants who they felt were in the greatest need of their support, and therefore, a selection bias cannot be excluded. Despite these limitations, this trial includes a robust sample of MDR-TB patients, one of the largest trials of MDR-TB patients published and the first NCM trial to demonstrate an intervention effect on treatment outcomes.

## CONCLUSION

Reducing treatment failure through a focused NCM intervention likely improved adherence and prevented lapses in treatment due to better patient engagement to support side effect management. These are hallmarks of a patient-centred care model that are easily scalable across TB programmes. Future research in this area should consider mechanisms such as cash transfer to address significant barriers associated with poverty or decentralised models that create treatment proximity to the patient’s home to reduce distance to treatment.

## Supplementary Material
