# Supplementary material for: Nurse case management to improve multidrug-resistant TB: a cluster-randomised trial
Source: IJTLD Open. 2026 Jul 13;3(7):444–52. doi: 10.5588/ijtldopen.26.0126 (PMC13362299; doi:10.5588/ijtldopen.26.0126)
Supplement: Supplementary file 1 [file ijtldopen26-0126_supplementarydata1.pdf]

Table S1: NCM Intervention Organized According to Chronic Care Model

Delivery System	Care coordination across inpatient facilities (i.e. linkage to care)<br>a. Evaluate readiness for discharge to down referral or outpatient management<br>b. Referral to supportive services (audiology, physiotherapy, nutritionist and palliative care as necessary)<br>c. Active case finding of household contacts through text message contacts and home visits
Delivery System	Care coordination across primary healthcare and hospital outpatient facilities (i.e. linkage to care)<br>a. Monitor adherence to MDR-TB and HIV visits<br>b. Identify missed appointments and assign tracing team if patient cannot be found<br>c. Provide an ongoing resource for patients within the community via telephone triage of problems
Decision Support	MDR-TB and HIV regimen selection<br>a. Support guideline-based regimen selection and laboratory testing (guidelines followed)<br>b. Monitor changes to treatment regimens (changes implemented when necessary)<br>c. Time to ART initiation after MDR-TB hospital admission
Decision Support	HIV and chronic disease integration: test, refer, treat strategy<br>a. Initiate HIV counseling and testing<br>b. Refer to ART counselor, as necessary (linkage to care)<br>c. Evaluation for first window of opportunity to begin ART after MDR-TB treatment initiation
Decision Support	HIV integration: opportunistic infection (OI) prevention screening & initiation<br>a. Monitor initiation of cotrimoxazole (CPT)<br>b. Appropriate OI and concomitant illness screening
Decision Support	Active surveillance for adverse drug reactions (ADRs)<br>a. Active monitoring for ADRs<br>b. Communicate event with physician<br>c. Develop plan to remedy ADR in consultation with physician
Clinical Information Systems	Coordination of MDR-TB/HIV monthly case discussion<br>a. Selection of patients and coordination of clinical review with HIV team, if applicable
Clinical Information Systems	Interdisciplinary communication<br>a. Completion of interdisciplinary weekly goal sheet during inpatient rounds
Patient Self-Support	Patient coaching/support/skills building to encourage medication adherence<br>a. Provide education and anticipatory guidance for maintaining adherence<br>b. Encourage self-report of difficulties with adherence
Community Resources	Evaluation of psychosocial community needs<br>a. Identify barriers to care and plan to remedy<br>b. Arrange parental/guardian transport for visitation of admitted children (13-17 years)

MDR-TB: multidrug resistant tuberculosis; HIV: human immunodeficiency virus; ART: antiretroviral treatment; CPT: cotrimoxazole preventive treatment
